# Respiratory Biofeedback Does Not Facilitate Lowering Arousal in Meditation Through Virtual Reality

**DOI:** 10.1007/s10484-018-9421-5

**Published:** 2018-10-30

**Authors:** Angelica M. Tinga, Ivan Nyklíček, Michel P. Jansen, Tycho T. de Back, Max M. Louwerse

**Affiliations:** 1 Department of Cognitive Science & Artificial Intelligence, Tilburg University, Dante Building, Room D 330, Warandelaan 2, 5037 AB Tilburg, The Netherlands; 2 Department of Medical and Clinical Psychology, Tilburg University, Tilburg, The Netherlands; 3 Department of Electrical Engineering, Mathematics, and Computer Science, University of Twente, Enschede, The Netherlands

**Keywords:** Meditation, Biofeedback, Virtual reality, Arousal, Respiration, Electroencephalography

## Abstract

The current study examined the effectiveness of respiratory biofeedback in lowering subjective and objective arousal after stress. Participants were presented with a meditation session in virtual reality while subjective and objective arousal were measured, the latter measured through ECG and EEG. Three conditions were used: (a) a respiratory biofeedback condition, in which visual feedback was paired to breathing; (b) a control feedback placebo condition, in which visual feedback was not paired to breathing; and (c) a control no-feedback condition, in which no visual feedback was used. Subjective and objective arousal decreased during meditation after stress in all conditions, demonstrating recovery after stress during meditation in virtual reality. However, the reduction in arousal (on all outcome measures combined and heart rate specifically) was largest in the control feedback placebo condition, in which no biofeedback was used, indicating that respiratory biofeedback had no additional value in reducing arousal. The findings of the current study highlight the importance of including a control feedback placebo condition in order to establish the exact additional value of biofeedback and offer insights in applying cost-effective virtual reality meditation training.

## Introduction

Anxiety and stress-related illnesses and disorders are among the most frequently encountered mental problems and are associated with substantial psychosocial and cognitive impairment (Wittchen et al. 2011; Baxter et al. 2013). At the heart of many evidence-based psychological therapies for stress-related illnesses and disorders are relaxation and breathing exercises, teaching people to breathe slowly and steadily through their diaphragm (Silverman et al. 2008; Kim et al. 2013). Recently it has been demonstrated that live auditory and/or visual feedback paired to breathing, also called respiratory biofeedback, is effective in breathing exercises (Yu et al. 2012; van Rooij et al. 2016; Giggins et al. 2013; Kaushik et al. 2005). In respiratory biofeedback breathing is measured using electrodes or sensors attached to the upper body and the breathing measures are converted to auditory and/or visual information that is presented to the user (Giggins et al. 2013). Respiratory biofeedback may have shown to be effective, yet it has the drawback of the necessity of using relatively expensive and intrusive equipment, diminishing the wide-spread use of relaxation and breathing exercises using biofeedback.

If studies on respiratory biofeedback and stress or anxiety included a control condition, it was one without any intervention (Yu et al. 2012; van Rooij et al. 2016; Kaushik et al. 2005; Kapitza et al. 2010). However, to assess the added value of respiratory biofeedback, a control placebo condition that is only different from a biofeedback condition regarding the specific biofeedback component is needed (Kapitza et al. 2010). In such a control feedback placebo condition collected breathing measures are not used in the presentation of auditory and/or visual information to the user. When such a condition was included in a study on respiratory biofeedback in patients with chronic back pain, there was no significant difference in pain levels, health and autonomic symptoms between patients that received biofeedback and patients that received control feedback placebo (Kapitza et al. 2010).

Relaxation and breathing exercises can benefit from virtual reality, a method increasingly applied in domains of therapy and rehabilitation (Bohil et al. 2011; Rizzo and Kim 2005). Advances in virtual reality technology and reductions in the associated costs make this tool more applicable, useful and accessible, providing promising possibilities for systematic testing, training, and treatment with precise control and measurements (Rizzo and Kim 2005). In the area of anxiety and stress-related illnesses and disorders virtual reality looks promising in treatment (Goncalves et al. 2012; Diemer et al. 2014).^1^ Preliminary research on combining virtual reality with respiratory biofeedback suggests this combination could be successful in reducing anxiety in children as measured by comparing pre- to post-levels of anxiety (van Rooij et al. 2016). However, as in non-virtual reality studies, no comparison between respiratory biofeedback and a control feedback placebo condition was made, making it difficult to determine the added value of respiratory biofeedback.

The current study aimed to remediate the critical issue of valid comparisons by examining the effectiveness of respiratory biofeedback during virtual reality meditation in lowering subjective and objective arousal after stress. Subjective arousal was measured through self-reports; objective arousal was measured through electrocardiography (ECG) and electroencephalography (EEG). Three different conditions during virtual reality meditation were presented: (a) a respiratory biofeedback condition in which visual feedback was paired to breathing; (b) a control feedback placebo condition in which visual feedback was not paired to breathing; and (c) a control no-feedback condition, in which no visual feedback was used. Based on studies demonstrating the effectiveness of respiratory biofeedback (Yu et al. 2012; van Rooij et al. 2016; Giggins et al. 2013; Kaushik et al. 2005) we expected the highest arousal reduction with respiratory biofeedback. However, when considering the finding that respiratory biofeedback does not have any added value compared to a control feedback placebo condition in patients with chronic back pain (Kapitza et al. 2010) we expected no difference in arousal reduction between respiratory biofeedback and control feedback placebo. In both cases we expected both the biofeedback and control feedback placebo conditions to reduce arousal more than the no feedback condition.

## Methods

### Participants

Sixty Tilburg University students (37 female), 20 in each of the three experimental conditions, received one course credit or 10 euros for their participation. Participants were between 18 and 31 years old (*M* = 22.07; *SD* = 3.03). Participants were included if they reported no current anxiety disorder, cardiovascular disease, neurological disorder, and lung disease. The study was approved by the Research Ethics Committee of Tilburg School of Humanities and Digital Sciences.

### Materials and Apparatus

#### Trier Social Stress Task

In order to induce stress in participants, a part of the Trier Social Stress Task (TSST) was used. The TSST is a standardized, validated and highly reliable protocol for experimentally inducing both subjective and objective physiological stress, based on more than 20 years of research (Kudielka et al. 2007; Allen et al. 2014). It starts with preparing and giving a presentation, followed by performing a mental arithmetic task. In the arithmetic task participants have to count backwards from a large number in steps of a certain order under time pressure. When an error is made, participants have to start over.

In the current study we presented participants with the mental arithmetic task part of the TSST. Mental arithmetic tasks are widely accepted as a mental stressor and show high test–retest correlations (Noto et al. 2005; Condren et al. 2002; Jern et al. 1991). Participants had to start counting backwards aloud from 1000 in steps of 13 as quickly and accurately as possible. They had 5 min to reach a number as low as possible, while the experimenter checked each answer provided by the participants. When a mistake was made, the experimenter interfered: “Stop–Mistake–Start over at 1000”. When a participant slowed down, the experimenter said: “You are quite slow, try to be quicker”. After participants reached 870 for the first time, the experimenter told every participant: “Currently you are performing worse than other participants”.

#### Meditation Task

The meditation task was presented in virtual reality via an Oculus Rift DK2 (resolution: 960 × 1080 pixels per eye; refresh rate: 75 Hz, head rotation locked). The task consisted of an audio guided meditation lasting 5 min and 48 s. Its instructions were based on the practice of Satipatthána, an integral part of Buddhist practices for achieving mindfulness (Thera 1941; Anālayo 2003). Mindfulness can be regarded as a process of attention regulation in order to focus on current experiences with curiosity, openness and acceptance (Bishop et al. 2004). The guided meditation focused on mindful breathing, by instructing to “focus on your breathing” and to “mindfully breathe in” and to “mindfully breathe out”, while “experiencing the whole body”. Participants were additionally stimulated to breathe deeply as the instructions indicated multiple times to “breathe deeper and deeper” and to “take a deep breath in, and release”, while the instructor breathed in and out deeply and slowly.

**Fig. 1:**
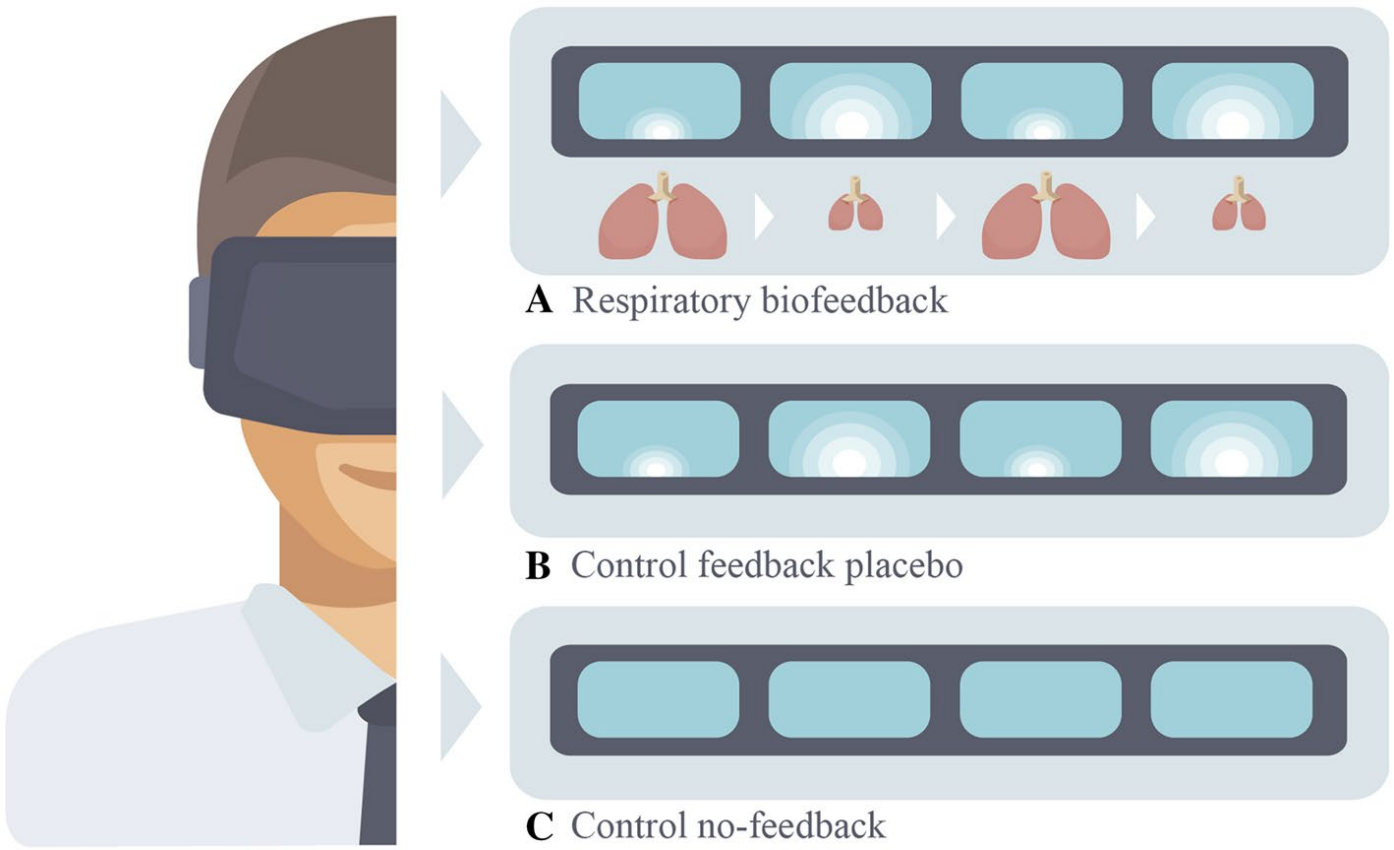
Schematic depiction of the three conditions. **a** respiratory biofeedback condition (visual feedback paired to breathing), **b** control feedback placebo condition (visual feedback not coupled to breathing), **c** control no-feedback condition (no visual feedback)

In the respiratory biofeedback condition and control feedback placebo condition participants saw a white cloud moving towards them in the direction of their mouth while the cloud got smaller and moving away from them while getting larger. In the respiratory biofeedback condition the movements of the cloud were controlled by the participant’s respiration with the cloud moving towards the participant while breathing in and away from the participant while breathing out. When participants were breathing in and out slowly and deeply, the virtual environment was filled completely by the cloud at the moment of complete exhalation in order to stimulate calm and deep breathing. In the control feedback placebo condition the movements of the cloud were automatic with the cloud moving towards the participant in 3 s and away from the participants in another 3 s [i.e. ten complete movements per minute, following Strauss-Blasche et al. (2000)], irrespective of the participants’ breathing. In the control no-feedback condition participants only saw a blue background. Figure 1 schematically depicts the three conditions.

#### Measures of Subjective Arousal

Following Littel et al. (2017) subjective tension and subjective calmness were measured with 10 cm visual analogue scales (VAS) ranging from 0 (*not tense/calm at all*) to 10 (*very tense/calm*). Results for subjective calmness were strongly correlated (Cohen 1988) to results for subjective tension, *r* = − .77, *p* < .001. Therefore, only subjective tension was included in the current study’s analyses.

#### Measures of Objective Arousal

Three-lead ECG and respiration (respiratory effort transducer, SS5LB, BIOPAC Systems, Inc.) were measured continuously throughout the experiment at 2000 samples per second using a BioNomadix wireless system (BN-RSPEC, BIOPAC Systems, Inc.). The ECG and respiration signals were bandlimited online from 0.05 to 150 Hz and from DC to 10 Hz respectively. These signals were collected by the software program AcqKnowledge 5.0 (BIOPAC Systems, Inc.) running on a computer solely dedicated to collecting the ECG and respiration data. ECG data were collected for further analyses, while respiration data were used online in the virtual reality simulation in the respiratory biofeedback condition and for offline visual inspection of the data in order to get insight into whether participants were following the meditation instructions.

Nine-channels (Fz, F3, F4, Cz, C3, C4, Pz, P3, and P4) EEG were measured continuously throughout the experiment at 256 samples per second using a wireless B-Alert X10 system (ABM). The EEG signals were collected by the software program AcqKnowledge 4.4 (BIOPAC Systems, Inc.) running on a computer solely dedicated to collecting the EEG data.

### Procedure

**Fig. 2:**
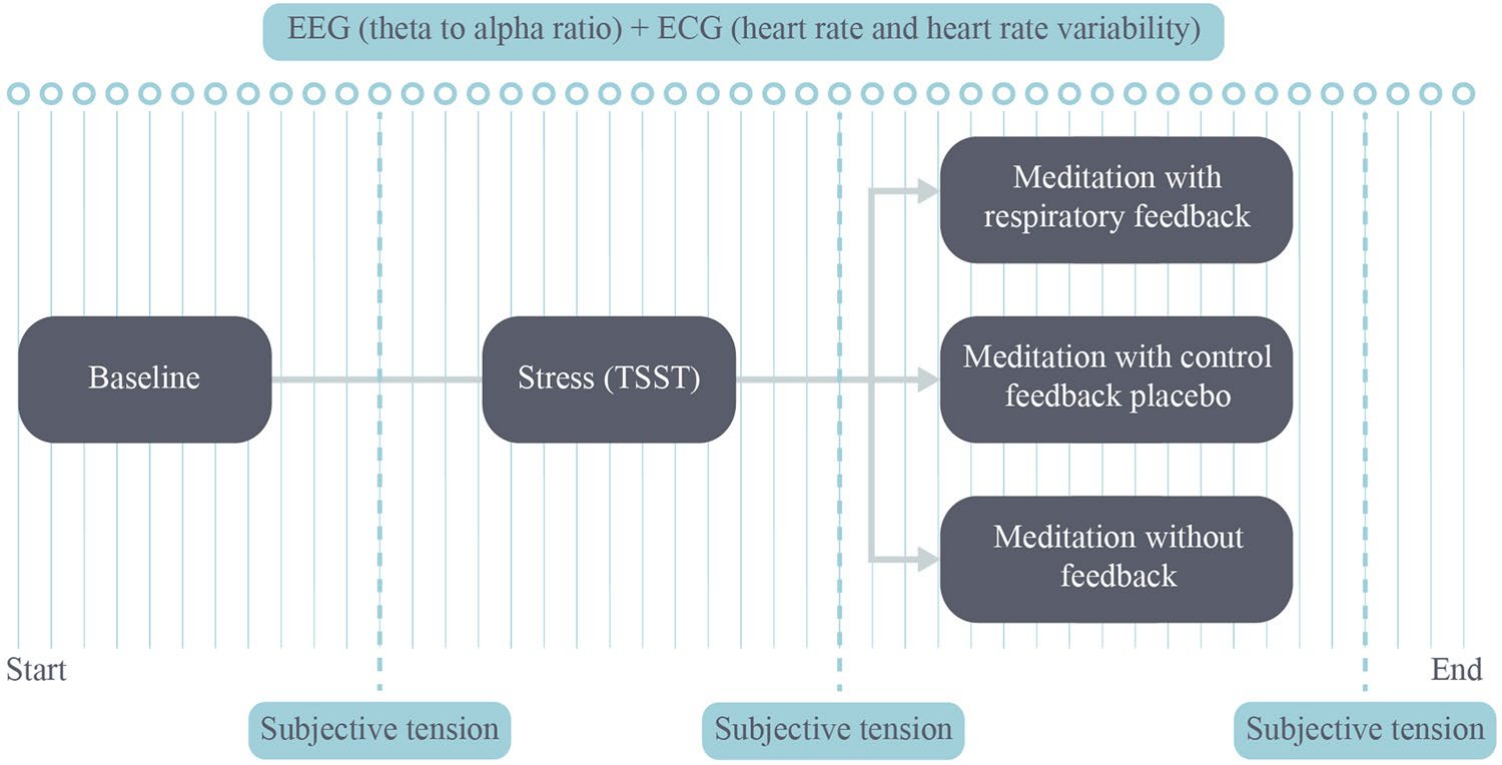
Diagram of the study design. Dark boxes with light text show the three phases of the experiment, light boxes with dark text show the measurements of subjective (collected at three time points) and objective arousal (collected continuously)

The design of the study is depicted in Fig. 2. After obtaining written and verbal informed consent, participants filled out a questionnaire on demographics. The EEG, ECG, and respiration sensors were placed on the participant and collection of these objective measures of arousal was started (Baseline). Afterwards, participants indicated how tense they felt. The arithmetic task component of the TSST was performed to induce arousal in participants before starting the meditation task (Stress). After completion of the TSST, subjective tension was measured for the second time.

Participants were assigned to the respiratory biofeedback condition, control feedback placebo condition or control no-feedback condition using block randomization in order to take individual difference factors into account and ensure an equal number of participants to be included in each condition (Goodwin 2009). All participants were instructed to follow the audio guided meditation instructions while keeping their eyes open. In the respiratory biofeedback condition and control no-feedback condition participants were also instructed that they would see a visualization in the virtual reality environment which they could use in any way they wanted to during the meditation. In the respiratory biofeedback condition they were additionally told that the visualization would change based on their breathing. Then, the meditation task started (Meditation). After finishing the meditation task, subjective tension was measured for the third and last time. Subsequently, participants were presented with a post-experiment questionnaire asking them whether they kept their eyes open during the meditation task and to indicate how pleasant the meditation task was and how well they could relax on the meditation task on 10 cm VAS scales ranging from 0 (*not pleasant/well at all*) to 10 (*very pleasant/well*). Finally, participants were debriefed and the sensors were removed.

### Data Processing and Analyses

#### Processing Objective Measures of Arousal

A finite impulse response (FIR) high-pass filter at 1 Hz and a FIR low-pass filter at 35 Hz were applied on the recorded ECG signal. QRS complexes were automatically identified in the ECG signal using AcqKnowledge 5.0. The identified complexes were manually checked and adjusted where needed. Based on the marked QRS complexes heart rate and the root mean square of successive differences (RMSSD) as a measure of heart rate variability were computed in AcqKnowledge 5.0.

The FieldTrip EEG processing toolbox (version 2015-10-20) (Oostenveld et al. 2011) was used in the software program MATLAB (version R2015a) to process the collected EEG signal. First, the quality check reports provided by FieldTrip were visually inspected for each individual participant to assess the quality of each data file. Data were low-passed filtered at 40 Hz and the theta (4–7 Hz) and alpha frequency (8–13 Hz) for each channel were computed with the multitaper method using Hanning tapers. Thereafter, the theta to alpha ratio was computed for each channel. Theta and alpha power are often used when it comes to evaluating the success of meditation (Chiesa 2009). The ratio between the two power bands was examined as theta power was shown to decrease and alpha power to increase during meditation focused on breathing (Park and Park 2012). This measurement was examined for all channels together, as effects on both theta and alpha activity during meditation are reported throughout the whole scalp (Kaur and Singh 2015).

#### Statistical Analyses

The average heart rate, heart rate variability and EEG theta to alpha ratio were computed for three time periods: (1) the minute preceding the start of the TSST (Baseline); (2) the 5 min during the TSST (Stress); and (3) the 5 min and 48 s during the meditation task (Meditation). The first measure of subjective tension was used as baseline, the second measure of subjective tension was used to measure the effect of the TSST (Stress) and the third measure of subjective tension was used to measure the effect of the meditation task (Meditation).

To determine whether the three conditions were not different before Meditation, a randomization check was performed for all included outcome measures using MANOVA testing the effect of condition at Baseline and Stress. Additionally, the effect of the TSST and the effect of meditation in virtual reality after stress was tested by comparing all outcome measures at Baseline to Stress and at Stress to Meditation respectively, using repeated measures MANOVA and post-hoc repeated measures ANOVAs on each individual outcome measure. Additionally, as the response to the stress manipulation might have had an effect on how strongly arousal decreased in the meditation task, the relationship between the change in arousal from Baseline to Stress and the change in arousal from Stress to Meditation was examined using Pearson correlation. In addition, we assessed whether there was no difference in this relationship between the three conditions by using MANOVA testing the effect of condition on the relationships’ residuals. In order to test the effect of the different conditions in the meditation task, difference scores were computed by subtracting Stress from Meditation. These difference scores of all outcome measures were entered in MANOVAs testing the general effect of feedback (respiratory biofeedback and control feedback placebo) versus no feedback (control no-feedback) on arousal reduction from Stress to Meditation and testing the difference between respiratory biofeedback and control feedback placebo. Post-hoc ANOVAs were conducted in order to examine effects on each separate outcome measure. As an exploratory analysis in order to gain more insight in the experience of the participants in the different conditions during the meditation task, the effect of condition on the indicated pleasantness of and indicated degree of ease to relax during the meditation task was analyzed using ANOVAs.

In all between-subjects analyses and in the correlation analyses we controlled for age and gender to account for individual differences as these factors are able to have an influence on neurophysiological outcomes (Tinga et al. 2018).

## Results and Discussion

All included participants indicated they kept their eyes open during the meditation task, with no indication after visual inspection of the respiration data of participants not following the instructions in the meditation task. Based on the visual inspection of Fieldtrip’s EEG quality check reports, EEG data of six participants were not included for further EEG analyses. Thus, data of 54 participants (18 participants in the respiratory biofeedback condition, 19 participants in the control feedback placebo condition, and 17 participants in the control no-feedback condition) were included for analyses including EEG data. For all other (non-EEG) analyses, data of all 60 participants (20 participants in each condition) were included.

### Randomization and Manipulation Tests

At Baseline and Stress, there were no significant differences between the three conditions on all included outcome measures, *F*(8, 92) = 0.95, *p* = .482 and *F*(8, 92) = 1.10, *p* = .369 respectively. This is not surprising, as conditions were not supposed to differ before the manipulations were applied, i.e., tests of randomization demonstrated that the randomization was successful.

Arousal significantly increased from Baseline to Stress, *F*(1, 159) = 31.46, *p* < .001, η_p_^2^ = 0.37. Post-hoc repeated measures ANOVAs demonstrated that this increase was significant for subjective tension, *F*(1, 59) = 76.75, *p* < .001, η_p_^2^ = 0.57 and heart rate, *F*(1, 59) = 11.60, *p* = .001, η_p_^2^ = 0.16, but did not reach significance for heart rate variability, *F*(1, 59) = 2.01, *p* = .161, η_p_^2^ = 0.03 and EEG theta to alpha ratio, *F*(1, 53) = 0.85, *p* = .362, η_p_^2^ = 0.02. Thus, the TSST induced stress as demonstrated by the effect on all outcome measures together and subjective tension and heart rate specifically.

Arousal significantly decreased from Stress to Meditation, *F*(1, 159) = 5.44, *p* = .001, η_p_^2^ = 0.10. Post-hoc repeated measures ANOVAs demonstrated that arousal decreased on all outcome measures, all *F*s ≥ 26.39, all *p*s ≤ .001 and all η_p_^2^s ≥ 0.31. These findings reflect recovery after stress during meditation in virtual reality on all outcome measures combined and separately.

For all outcome measures there was a significant negative relationship between the change in arousal from Baseline to Stress and the change in arousal from Stress to Meditation, all absolute *r*s ≥ .64, all *p*s ≤ .001. This relationship was not different between the three conditions, *F*(8, 92) = 1.77, *p* = .094. Thus, the larger the increase in arousal during the stress manipulation, the larger the decrease in arousal during the meditation task, irrespective of condition.

### Tests of Effect of Condition

#### Effect of Feedback Versus No Feedback

There was no difference in arousal reduction on all outcome measures combined from Stress to Meditation with feedback (respiratory biofeedback and control feedback placebo) compared to control no-feedback, *F*(4, 46) = 1.09, *p* = .375, η_p_^2^ = 0.09. Effects were also not significant when considering outcomes for subjective tension, *F*(1, 56) = 2.08, *p* = .155, η_p_^2^ = 0.04, heart rate, *F*(1, 56) = 0.20, *p* = .658, η_p_^2^ = 0.01, heart rate variability as measured by RMSSD, *F*(1, 56) = 0.02, *p* = .881, η_p_^2^ < 0.01 and EEG theta to alpha ratio individually, *F*(1, 49) = 0.14, *p* = .712, η_p_^2^ < 0.01.

These results indicated that it made no difference whether feedback (respiratory biofeedback and control feedback placebo) or no feedback was provided during virtual reality meditation after stress.

#### Effect of Respiratory Biofeedback Versus Control Feedback Placebo

**Fig. 3:**
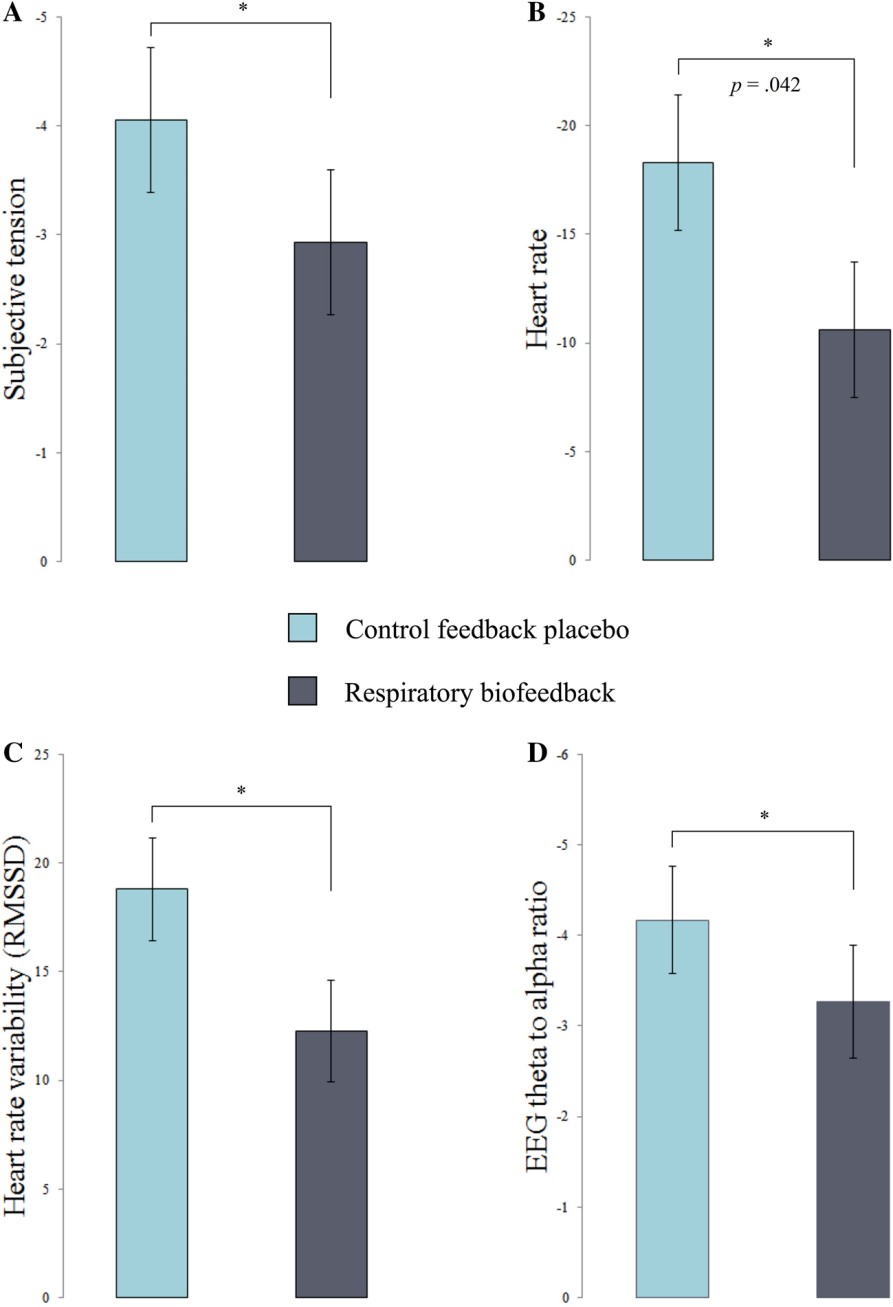
Bar charts depicting the change in subjective tension (**a**), heart rate (**b**), heart rate variability (**c**) and EEG theta to alpha ratio (**d**) from stress to meditation with control feedback placebo (in light color) and respiratory biofeedback (in dark color). Error bars represent SEM. Asterisks indicate the significant effect of respiratory biofeedback versus control feedback placebo for all outcome measures combined (*p* = .043). The *p* value indicates the additionally significant effect for heart rate specifically (*p* = .042)

Regarding the effect of respiratory biofeedback versus control feedback placebo, there was a significant difference in arousal reduction from Stress to Meditation on all outcome measures combined, *F*(4, 29) = 2.82, *p* = .043, η_p_^2^ = 0.28. As can be seen in Fig. 3, average arousal reduction for all outcome measures was higher with control feedback placebo than with respiratory biofeedback. Concerning subjective tension separately (Fig. 3A), however, there was no significant difference between these two different types of feedback, *F*(1, 36) = 1.14, *p* = .293, η_p_^2^ = 0.03. The difference between the two different types of feedback was significant for heart rate (Fig. 3b), demonstrating that estimated marginal means decreased with 7.39 (*SE* = 3.50) beats per minute more when receiving control feedback placebo than when receiving respiratory biofeedback, *F*(1, 36) = 4.45, *p* = .042, η_p_^2^ = 0.11. There was no significant effect when considering heart rate variability as measured by RMSSD (Fig. 3c) or the EEG theta to alpha ratio specifically (Fig. 3d), *F*(1, 36) = 0.76, *p* = .391, η_p_^2^ = 0.02 and *F*(1, 32) = 1.12, *p* = .299, η_p_^2^ = 0.03 respectively.

Taken together, these results indicated that control feedback placebo was superior to respiratory biofeedback in reducing arousal, as measured by all outcome measures together and heart rate specifically, during meditation in virtual reality after stress.

#### Subjective Experience During Meditation Task

Participants rated the pleasantness of the meditation task with an average of 6.24 (*SE* = 0.24) on a scale of 0–10. On average, participants rated the ease with which they could relax during Meditation with a 6.26 (*SE* = 0.27) on a scale of 0–10. There were no differences in ratings of pleasantness and ease of relaxation between feedback versus no feedback, *F*(1, 56) = 1.20, *p* = .278, η_p_^2^ = 0.02 and *F*(1, 56) = 0.04, *p* = .838, η_p_^2^ < 0.01 respectively and between respiratory biofeedback and control feedback placebo, *F*(1, 36) = 0.71, *p* = .404, η_p_^2^ = 0.02 and *F*(1, 36) = 3.26, *p* = .079, η_p_^2^ = 0.42 respectively. These results give no indication that subjective experiences during the meditation task were significantly different between conditions.

## General Discussion

The aim of the current study was to examine the effectiveness of respiratory biofeedback during virtual reality meditation in lowering subjective (subjective tension) and objective arousal (EEG and heart rate [variability]) after stress. Visual feedback paired to participants’ breathing (respiratory biofeedback) was compared to a control feedback placebo condition in which visual feedback unpaired to participants’ breathing was presented and a control condition in which no feedback was provided at all. Subjective and objective arousal decreased during meditation after stress in all conditions. This is in line with previous research demonstrating that virtual reality is effective in relaxation and breathing exercises (Bohil et al. 2011; Rizzo and Kim 2005; van Rooij et al. 2016).

However, contrary to what is suggested by other studies, respiratory biofeedback was not the most effective in reducing arousal: the reduction in arousal (on all outcome measures combined and heart rate specifically) in the current study was largest in the control feedback placebo condition, indicating that respiratory biofeedback had no additional value in reducing arousal and was even less effective than control feedback placebo. This current finding is in line with the finding of Kapitza et al. (2010) showing no preference for respiratory biofeedback compared to control feedback placebo in lowering pain levels in patients with chronic back pain. Additionally, the current finding suggests that preliminary research demonstrating that combining virtual reality with respiratory biofeedback is effective in reducing anxiety in children (van Rooij et al. 2016) should be interpreted with caution. As biofeedback in earlier work was not compared with comparable control feedback placebo not coupled to physiology, the possibility is left open that anxiety reduction could be comparable or even stronger without using biofeedback.

The current findings are important in at least two respects. First, the findings highlight the importance of including a control feedback placebo condition when studying the effectiveness of biofeedback in order to establish the exact additional value of providing biofeedback. Yet, to date, there is only a single study (Kapitza et al. 2010) that included a control feedback placebo condition in examining the role of biofeedback. Second, these findings suggest that if virtual reality is used for meditation, no biofeedback equipment is needed to reduce arousal, providing a more affordable and less intrusive option to applying virtual reality to relaxation exercises.

Future studies should examine the effectiveness of biofeedback compared to proper control conditions in different groups of people under different types of circumstances to determine exactly when and why biofeedback might be (un)preferable. For example, effects of biofeedback in children as examined in the study by van Rooij et al. (2016) might be different from effects of biofeedback in adults as examined in the current study. Another interesting direction for future work would be to examine the generalizability of biofeedback and other types of feedback, as it has been demonstrated for example that respiratory biofeedback is able to reduce respiration rate while leaving other neurophysiological outcomes (brain activity, heart rate, temperature and skin conductance) unaffected (Montgomery 1994). Examining the effectiveness of different types of control feedback placebos in order to establish the most effective one might additionally be an interesting focus for future research. For example, using control feedback at a rate that is similar to the resonance frequency as used in heart rate variability biofeedback (generally about six cycles per minute) might induce an even stronger reduction in arousal than the current study’s control feedback (ten cycles per minute) (Steffen et al. 2017; Lehrer and Gevirtz 2014). Determining the most effective control feedback placebo is especially interesting as employing this type of feedback would make an application aimed at reducing arousal easier to apply and more cost-effective than employing biofeedback. This would, in turn, ensure that more people suffering from anxiety and stress-related illnesses and disorders get the opportunity to benefit from those types of applications. Yet, for now, we have to conclude that respiratory biofeedback does not facilitate lowering arousal in meditation through virtual reality compared to control feedback placebo and no feedback at all.

## Footnotes

1 However, see a review of McCann et al. (2014) for some concerns regarding the quality of RCT studies in this area.
